# Pneumonia in trauma patients

**DOI:** 10.1186/2047-2994-4-S1-P242

**Published:** 2015-06-16

**Authors:** HIG Giamberardino, B Schelmezer, HZ Adamante, T Betim, M Ducroquet

**Affiliations:** 1 Epidemiology and Infection Control Center, Hospital do Trabalhador, Curitiba, Brazil

## Introduction

Ventilator associated pneumonia (VAP) is the main infection in multiple trauma patients in the Intensive Care Unit (ICU). The invasive treatment and clinical trauma conditions of these patients increase the risk of infections.

## Objectives

To study the incidence, etiology and risk factors associated with the development of ventilator associated pneumonia (VAP) in trauma patients admitted in an ICU.

## Methods

The study is a retrospective cohort study, of patients who developed VAP during hospitalization in a Trauma ICU, on a referral trauma center, with 220 beds, the Hospital do Trabalhador (HT). The study period was between January 2012 from December 2013. Diagnosis criteria of Centers for Disease and Control (CDC) were used. We developed an active search record, which was filled with information from medical records and the database of the Epidemiology and Infection Control Center from HT.

## Results

A total of 969 patients were ben hospitalized in ICU in the study period, and 125 were diagnosed with Ventilator Associated Pneumonia (VAP). Usage rate of ventilator was 58.32%. The sample was composed of males (79.2%), with ages ranging from 18 to 94 years, with an average of 42.7 years. The main mechanism of trauma was the automotive (48%), with the most prevalent being Traumatic Brain Injury (TBI) with 54.4% of the cases followed by the thoracic (35.2%). The mean ICU stay was 26.6 days (SD 21.8) and on mechanical ventilation (MV) 19.4 days (SD 17.7). The prevalent bacterial etiological agents of pneumonia were MSSA and MRSA, both with 21.6% followed by *Acinetobacter baumanii* (16.8%) and *Pseudomonas aeuroginosa* (16%). The therapy choice was piperacillin-tazobactam in 96 patients, corresponding to 58.4% of the sample.

## Conclusion

The incidence of PAV on trauma was associated with car accident and TBI, which is an independent predictor of mortality risk. Yet there was association between length of stay in ICU with PAV, and found a higher rate of patients with positive blood cultures in comparison with literature due to trauma patients have higher risk for sepsis.

## Disclosure of interest

None declared.

